# Analysis of terminal duct lobular unit involution in luminal A and basal breast cancers

**DOI:** 10.1186/bcr3170

**Published:** 2012-04-18

**Authors:** Xiaohong R Yang, Jonine D Figueroa, Roni T Falk, Hong Zhang, Ruth M Pfeiffer, Stephen M Hewitt, Jolanta Lissowska, Beata Peplonska, Louise Brinton, Montserrat Garcia-Closas, Mark E Sherman

**Affiliations:** 1 Division of Cancer Epidemiology & Genetics, National Cancer Institute, National Institutes of Health, Bethesda, MD, USA; 2 Institute of Biostatistics, School of Life Science, Fudan University, Shanghai, China; 3 Tissue Array Research Program, Laboratory of Pathology, Center for Cancer Research, National Cancer Institute, National Institutes of Health, Bethesda, MD, USA; 4 Department of Cancer Epidemiology and Prevention, Cancer Center and M. Sklodowska-Curie Institute of Oncology, 02-781 Warsaw, Poland; 5 Nofer Institute of Occupational Medicine, 91-348 Lodz, Poland; 6 Division of Genetics and Epidemiology, Institute of Cancer Research, London, UK

## Abstract

**Introduction:**

Involution of terminal duct lobular units (TDLUs), the structures that give rise to most breast cancers, has been associated with reduced breast cancer risk. Data suggest that the etiology and pathogenesis of luminal A and core basal phenotype (CBP) breast cancers differ, but associations with TDLU involution are unknown. Accordingly, we performed a masked microscopic assessment of TDLU involution in benign tissues associated with luminal A and CBP breast cancers diagnosed among women less than age 55 years.

**Methods:**

Cases were participants in a population-based case-control study conducted in Poland. Increased TDLU involution was defined as fewer acini per TDLU or shorter TDLU diameter. Luminal A was defined as estrogen receptor (ER) positive and/or progesterone receptor (PR) positive and human epidermal growth factor receptor 2 (HER2) negative and CBP as negative for ER, PR, and HER2 with expression of basal cytokeratins or epidermal growth factor receptor (EGFR). We performed logistic regression to evaluate associations between TDLU involution and tumor subtypes, adjusted for clinical characteristics and breast cancer risk factors.

**Results:**

Among 232 luminal A and 49 CBP cancers associated with evaluable TDLUs, CBP tumors were associated with significantly greater average number of acini per TDLU (odds ratio (OR) = 3.36, 95% confidence interval (CI) = 1.36 to 8.32, *P *= 0.009) and larger average TDLU diameter (OR = 2.49, 95% CI = 1.08 to 5.74, *P *= 0.03; comparing highest to lowest group, adjusted for age and study site).

**Conclusions:**

We suggest that TDLU involution is less marked in benign tissues surrounding CBP as compared to luminal A cancers, which may reflect differences in the etiology and pathogenesis of these tumor subtypes.

## Introduction

Epidemiologic research has demonstrated that risk factor associations for breast cancer vary by estrogen receptor (ER) status [1-3]. Recent studies that have included more detailed characterization of tumor markers suggest that differences in risk factor associations between luminal A cancers (ER and/or progesterone receptor (PR) positive and human epidermal growth factor receptor 2 (HER2) negative) and core basal phenotype (CBP) cancers ("triple negative" for ER, PR and HER2 with expression of basal cytokeratins or epidermal growth factor receptor (EGFR)) account for much of this etiological heterogeneity. In a pooled analysis of 12 population-based studies included in the Breast Cancer Association Consortium, risk for luminal A cancers was inversely associated with having had a live birth, younger age at first full-term birth, and premenopausal obesity, whereas these factors were not associated with risk for CBP cancers [4]. In contrast to luminal A cancers, CBP cancers occur more often among African Americans, *BRCA1 *mutation carriers and younger women, providing additional support for the view that these tumors are etiologically different [5,6].

Luminal A and CBP cancers are distinguished by their gene expression and immunohistochemical profiles [7-9]. These tumors may arise from different stem or progenitor cells [10-12] and thus may develop via pathways that diverge early in tumor development. In support of this view, data suggest that the molecular characteristics of benign tissue surrounding specific breast cancer subtypes may be as distinctive as that of cancer tissue itself. Specifically, invasive breast cancers arise from terminal duct lobular units (TDLUs), and data suggest that the mRNA expression profiles of micro-dissected TDLUs surrounding ER positive and ER negative breast cancers differ [13]. Other studies have found that mRNA expression in benign tissues associated with CBP cancers demonstrate a "wound signature", which seems to reflect the characteristic biology of these tumors (that is, epithelial mesenchymal transition) [14]. Although molecular analyses of peritumoral tissues are providing evidence for breast field effects (the presence of larger areas of molecular alteration surrounding histologically confirmed breast cancer foci), epidemiological data inter-relating breast cancer risk factors, breast cancer subtypes and characteristics of benign tissues surrounding cancers are limited.

TDLU involution, as assessed morphologically, has been hypothesized to be associated with breast cancer risk [15]. Analyses performed in the Mayo Clinic Benign Breast Disease cohort and the Nurses' Health Study support this view [3,16]. We extend this hypothesis by proposing that the pattern of TDLU involution, as defined by acini per TDLU or TDLU diameter, over the life course differs in breasts containing luminal A vs. CBP breast cancers. Accordingly, we performed an exploratory masked histological analysis of TDLU involution using benign tissues collected in a population-based breast cancer case-control study conducted in Poland, which included detailed assessment of breast cancer risk factors and pathologic characteristics.

## Materials and methods

### Study population

Data and biologic specimens for the current analysis came from a population-based breast cancer case-control study conducted in Warsaw and Łodz, Poland from 2000 to 2003, as previously described in detail [17]. Eligible cases were women between the ages of 20 and 74 years diagnosed pathologically with incident *in situ *or invasive breast carcinoma. Control subjects were randomly selected using a population-based database, frequency-matched to cases on city and age in five-year categories. A total of 2,386 cases (79% of eligible) and 2,502 controls (69% of eligible) agreed to participate in the study and provided informed consent. The study was approved by the National Cancer Institute and local Institutional Review Boards in Poland. Since the goal of the current study was to compare TDLU involution between tumor subtypes, controls were not included in the analysis.

At the time of routine pathological evaluation, we collected one tumor block (T) and two "grossly" benign tissue blocks, from peritumoral (PT; adjacent to but not touching the tumor) and distant regions from the tumors (DT; at the periphery of the specimen) when possible. We restricted the current analysis to luminal A and CBP cancers because these tumors are viewed as most divergent with regard to etiology and pathogenesis. Benign tissue blocks (PT or DT) were available for 804 cases having these two tumor subtypes (83% of total luminal A and CBP cases). Tumor characteristics and risk factors did not differ significantly among cases with and without benign tissue blocks. We further restricted our analysis to 385 cases younger than age 55 years (317 luminal A and 68 CBP) because at older ages, TDLUs are often not identifiable in tissue sections, particularly in the absence of exogenous hormone use or obesity (data not shown).

### Risk factor assessment

We assessed breast cancer risk factors through a detailed personal interview as described elsewhere [17,18]. Factors evaluated included education, age at menarche, age at menopause, parity, breastfeeding, measured body mass index (BMI), age at first full-term birth, smoking, alcohol drinking, hormone replacement therapy (HRT) use among postmenopausal women, and family history of breast cancer.

### Pathology of cases

We assessed tumor size, histologic type, grade, and axillary lymph node status via clinical reports and independent review (MES) [17].

Procedures used for construction of tissue microarrays (TMAs) from invasive cancer tissues, immunohistochemical staining and scoring are described in detail elsewhere [18,19]. Previously, we assessed the expression of ER, PR, HER2, EGFR and cytokeratin 5 (CK5) using three different methods: (i) clinical reports of ER and PR (ii) immunohistochemical staining for cases included in TMAs, and (iii) AQUA™(HistoRx, Branford, CT), which is a quantitative immunofluorescent method that provides continuous measurement of expression levels [20]. Different measurement techniques showed strong agreement as previously reported [19]. For the current analysis, we classified tumors as luminal A (ER+ and/or PR+, HER2-) or CBP (ER-, PR-, HER2-, CK5+, and/or EGFR+) as previously described [18].

### Assessment of Terminal Duct Lobular Unit (TDLU) involution

One pathologist (MES) microscopically reviewed hematoxylin and eosin stained sections of benign blocks to assess characteristics of TDLUs, masked to all clinical and epidemiologic data, including tumor marker expression and subtype classification. To determine which TDLUs were suitable for assessment, we applied a modification of criteria proposed by Milanese *et al. *[16]. Specifically, we assessed TDLUs which: 1) consisted of acini lined by single luminal and myoepithelial cell layers; 2) displayed a limited extent of metaplasia or dilatation of acini (we excluded TDLUs in which 50% or more of the acini showed lumina three times the normal diameter or metaplastic epithelium), and 3) did not display benign breast disease, such as adenosis or other entities as implied from criteria above. However, we did not exclude TDLUs based on epithelial cell size *per se *or number of acini. Accordingly, some acini included in this analysis resemble the hyperplastic enlarged lobular units illustrated by Lee *et al. *[21].

**Figure 1:**
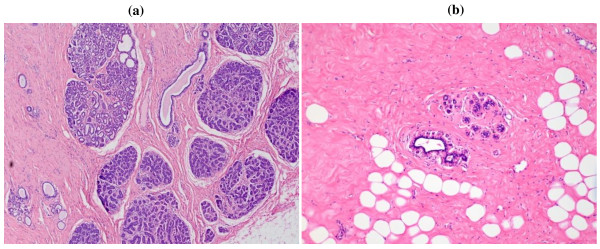
**TDLU images**. **A: **Minimal or no TDLU involution, characterized by densely clustered acini (magnification 5×); **B: **Marked TDLU involution demonstrating TDLUs containing few acini surrounded by dense collagen (magnification 10×).

We focused on two agnostic metrics of involution: average number of acini per TDLU and average TDLU diameter (both inversely related to involution). For each slide, we recorded the presence or absence of TDLUs; TDLU "diameter", defined as the maximal linear span of epithelium (relative to 400 × microscopic fields, approximated in 0.25 increments) and number of acini per TDLU (0 to 10, 11 to 20, 21 to 30, 31 to 50, 51 to 100, > 100) (Figure 1). We first conducted a pilot study of 200 slides and measured all identifiable TDLUs present (> 1,000). The average number of acini per TDLU and TDLU diameters across all measured TDLUs did not vary significantly when six or more TDLUs were evaluated (Additional file 1, Figure S1). For the full review, we microscopically reviewed slides beginning at one end and sequentially categorizing up to 10 unselected TDLUs masked to other data.

After completing the slide review, we performed a masked repeat review of 30 randomly selected slides to assess reproducibility; ratings were significantly correlated (number of acini per TDLU, concordance = 77%, *P *= 0.002; TDLU diameter, concordance = 71%, *P *= 0.008). TDLU involution variables were not significantly different in PT and DT blocks (*P *> 0.1 for pair-wise differences between PT and DT for both number of acini per TDLU and TDLU diameter); therefore, we took the average values from PT and DT blocks for each TDLU variable if both were available. As expected, the average number of acini per TDLU and the average TDLU diameter showed strong correlation (r = 0.86, *P *< 0.0001).

### Statistical analyses

To assess whether TDLU involution varied by molecular characteristics of the cancers, we evaluated the relationships between immunohistochemical status of ER, PR, CK5 and EGFR in tumors (explanatory variables) and the average number of acini per TDLU or the average TDLU diameter (outcome variables) using likelihood ratio tests and unconditional polytomous logistic regression. We defined tertiles of the distribution of the average number of acini per TDLU and the average TDLU diameter using cut-points based on distributions among luminal A tumors. Results, based on using maximal, median and average values across TDLUs, were similar and, therefore, we present only the average values. We also compared continuous AQUA scores for ER, PR, EGFR and CK5 expression in invasive tumors [19], to the number of acini per TDLU or TDLU diameter categories using the Kruskal-Wallis test. To assess our main hypothesis, we evaluated whether the level of TDLU involution varied between luminal A and CBP cancers using adjusted logistic regression models with tumor subtype (CBP vs. luminal A) as the outcome variable and number of acini per TDLU or TDLU diameter as explanatory variables. Adjustment variables included age (five-year interval), study site, breast cancer risk factors (education, age at menarche, age at menopause, parity, age at first full-term birth, breastfeeding, HRT use among post-menopausal women, BMI, and family history of breast cancer). To evaluate which of several correlated tumor features (tumor size, histology, grade, and nodal status, tumor subtype) were most important in driving the associations with TDLU involution variables, we also fitted regression models with average acini per TDLU or average TDLU diameter as the outcome variable and tumor characteristics as the explanatory variables.

We used SAS (version 9.1.3, SAS Institute, Inc., Cary, NC, USA) software for all analyses.

## Results

### Characteristics of study population

Among the 385 cases younger than age 55 years, TDLUs were identified in 281 cases (232 luminal A and 49 CBP). Distributions of risk factors and pathologic characteristics did not differ significantly among cases with and without evaluable TDLUs, overall or within subtype (Additional file 1, Table S1).

### Relationships of number of acini per TDLU and TDLU diameter to individual tumor markers

First, we compared TDLU involution characteristics in tumor subtypes defined by individual markers (ER, PR, CK5, and EGFR), dichotomously categorized as negative or positive, and adjusted for age and study site. Compared with ER negative cases, ER positive cases were associated with a significantly reduced number of acini per TDLU (odds ratio (OR) = 0.34, 95% confidence interval (CI) = 0.17 to 0.70, *P *= 0.003, comparing the highest to the lowest group), whereas EGFR positive tumors were associated with a greater number of acini per TDLU than EGFR negative tumors (OR = 2.78, 95% CI = 1.21 to 6.39, *P *= 0.02). CK5 positive cancers were also associated with greater number of acini per TDLU than CK5 negative cancers, but the comparison was not statistically significant. The number of acini per TDLU was not associated with PR expression, tumor size, histological type, grade or axillary node status (Table 1). Results for TDLU diameter were similar to those for the number of acini per TDLU (for ER: OR = 0.38, 95% CI = 0.19 to 0.76, *P *= 0.006; for EGFR: OR = 2.95, 95% CI = 1.24 to 7.03, *P *= 0.01; comparing the highest to the lowest group).

**Table 1:** Relationship between average number of acini per TDLU and tumor characteristics among women < 55 years old.

	Average number of acini (tertiles)*							
	
Tumor characteristics	Low		Middle		High		OR (95% CI)**	P**
	**N**	**%**	**N**	**%**	**N**	**%**		
Tumor size								
≤ 2 cm	48	31.8	50	33.1	53	35.1	Ref	
> 2 cm	40	31.0	40	31.0	49	38.0	1.15 (0.64, 2.06)	0.64
Axillary node metastases								
Negative	54	34.2	47	29.7	57	36.1	Ref	
Positive	32	27.1	41	34.8	45	38.1	1.31 (0.72, 2.38)	0.37
Histology								
Ductal	49	27.5	56	31.5	73	41.0	Ref	
Lobular	18	40.9	14	31.8	12	27.3	0.45 (0.20, 1.03)	0.06
Mixed	11	30.6	17	47.2	8	22.2	0.47 (0.17, 1.27)	0.13
Other	10	43.5	3	13.0	10	43.5	0.79 (0.30, 2.07)	0.62
Tumor grade								
Well differentiated	25	36.2	19	27.5	25	36.2	Ref	
Moderately differentiated	43	28.5	54	35.8	54	35.8	1.06 (0.56, 2.13)	0.88
Poorly differentiated	20	32.8	17	27.9	24	39.3	0.97 (0.42, 2.25)	0.95
ER								
Negative	14	17.5	27	33.7	39	48.8	Ref	
Positive	74	36.8	63	31.3	64	31.8	0.34 (0.17, 0.70)	0.003
PR								
Negative	24	27.9	28	32.6	34	39.5	Ref	
Positive	64	32.8	62	31.8	69	35.4	0.76 (0.40, 1.43)	0.39
CK5								
Negative	72	34.6	64	30.8	72	34.6	Ref	
Positive	16	22.9	24	34.3	30	42.9	1.72 (0.85, 3.49)	0.13
EGFR								
Negative	79	34.1	78	33.6	75	32.3	Ref	
Positive	9	19.1	10	21.3	28	59.6	2.78 (1.21, 6.39)	0.02

*Number of acini reflects averaged values from TDLUs per case in tertiles from lowest to highest; Number of acini per TDLU (1: 0 to 10, 2: 11 to 20, 3: 21 to 30, 4: 31 to 50, 5: 51 to 100, 6: > 100), low = 1 to 1.34; middle = 1.34 to 1.8; high = > 1.8. **P-values, ORs and 95% CIs were obtained from logistic regression analyses comparing cases with the highest to the lowest tertile of number of acini per TDLU adjusted for age and study site.

**Figure 2:**
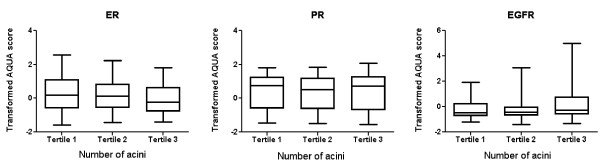
**Average number of acini per TDLU and marker expression levels in invasive tumors**. ER, PR, and EGFR expression levels were measured using AQUA™, which is a quantitative immunofluorescent method that provides continuous measurement of expression levels. AQUA scores were transformed as previously described [19].

Next, we evaluated relationships between TDLU involution characteristics and these tumor markers assessed as continuous variables. Analyses comparing number of acini per TDLU to continuous AQUA scores for ER, PR, and EGFR expression demonstrated associations consistent with those found in analyses in which markers were classified as negative or positive (Kruskal-Wallis test, *P *= 0.02 for ER and EGFR, *P *= 0.74 for PR, Figure 2).

### Number of acini per TDLU and TDLU diameter in luminal A vs. Core Basal Phenotype (CBP) cancers

The number of acini per TDLU and TDLU diameter were significantly greater for CBP as compared with luminal A tumors (Table 2). The associations remained significant after adjustment for age and study site (number of acini per TDLU: OR = 3.36, 95% CI = 1.36 to 8.32, *P *= 0.009; TDLU diameter: OR = 2.49, 95% CI = 1.08 to 5.74, *P *= 0.03; comparing the highest to the lowest group). Associations were equivalent or stronger in analyses adjusted for multiple breast cancer risk factors (education, age at menarche, age at menopause, parity, age at first full-term birth, breastfeeding, HRT use among post-menopausal women, BMI and family history of breast cancer; for number of acini per TDLU: OR = 4.44, 95% CI = 1.58 to 12.51, *P *= 0.005) (Table 2). When analyzing the correlated tumor characteristics simultaneously, the associations with both number of acini per TDLU and TDLU diameter were only observed for tumor subtype (CBP vs. luminal A) but not for tumor grade, histology, size, or nodal status (data not shown).

**Table 2:** TDLU involution variables and tumor subtypes (CBP vs.

Involution	Luminal A		CBP							
	
	N	%	N	%	OR*	95% CI	*P*	ORadj**	95% CI	*P*
#acini/TDLU										
Tertile 1	81	34.9	7	14.3	Ref			Ref		
Tertile 2	74	31.9	16	32.6	2.55	0.98, 6.64	0.05	3.08	1.07, 8.89	0.04
Tertile 3	77	33.2	26	53.1	3.36	1.36, 8.32	0.009	4.44	1.58, 12.51	0.005
										
Size of TDLUs										
Tertile 1	76	32.8	9	18.4	Ref			Ref		
Tertile 2	79	34.0	12	24.5	1.25	0.49, 3.16	0.64	1.35	0.47, 3.84	0.58
Tertile 3	77	33.2	28	57.1	2.49	1.08, 5.74	0.03	3.84	1.45, 10.13	0.007

Luminal A) among women < 55 years old.

## Discussion

Studies have found that reduced TDLU involution is an independent risk factor for breast cancer among women biopsied for benign breast disease [3,16,22]. Herein, we report results of a case-case comparison, showing that cancers that are ER negative (as compared to ER positive) or EGFR positive (as compared to EGFR negative) are associated with a greater number of acini per TDLU and larger TDLU diameters, consistent with reduced involution. Furthermore, based on immunohistochemical tumor subtyping, we found that TDLUs associated with CBP cancers are less involuted than those surrounding luminal A cancers, even following adjustment for age and other factors, including reproductive variables, BMI, family history of breast cancer and clinical variables. Our data are consistent with other studies showing that gene expression profiles of TDLUs surrounding cancers vary by the ER status of the tumor [13] and experimental evidence that stromal-epithelial interactions differ for luminal A and CBP cancers [23]. Further studies are needed to confirm these results and to determine whether these findings reflect differences in the etiology and pathogenesis of luminal A and CBP cancers or a secondary influence of tumor growth on surrounding benign tissues.

Prior studies have found that TDLU involution is directly associated with older age and inversely related to parity and early age at menarche. Although parity is associated with lower overall breast cancer risk, breast cancer risk increases transiently following pregnancy. In addition, parity-related breast cancer risk has also been associated with young age, having ER-negative tumors, late first full-term births, and failure to breastfeed [24,25]. Mechanisms that may contribute to the development of pregnancy-related cancers include exposure to elevated hormones during pregnancy and inflammation related to postpartum involution [24]. Data also suggest that pregnancy may induce proliferation of progenitor cells expressing basal markers [26], whereas long-term breastfeeding, which reduces cancer risk, may decrease the number of these cells by inducing terminal differentiation [26,27]. Therefore, a full-term pregnancy followed by a short duration of lactation may lead to retention of progenitor cells and increased risk for CBP cancers. Consistent with these findings, Symmans *et al. *found that women who do not breastfeed for longer periods are at increased risk of triple-negative breast cancers [28]. In a recent study of African American women with breast cancer, higher parity was associated with an increased risk of ER-negative or PR-negative breast cancer, which was mitigated by breastfeeding [29]. In this respect, our finding that TDLU involution is less in CBP as compared to luminal A cancers is consistent with the hypothesis that CBP cancers are related to hyperplastic processes in the breast that have not fully regressed postpartum.

Our findings may also have potential implications for understanding racial disparities in breast cancer. Compared to Caucasian women, African American women undergo menarche at younger ages [30], have higher parity [31], breastfeed less [6], and have higher rates of CBP cancers. Thus, etiological differences between luminal A and CBP breast cancers may reflect the effect and/or the interaction of risk factors on TDLU involution, molecular characteristics and function. Expansion of these results would have potential implications for developing prevention strategies because TDLU size could represent a biomarker for assessing breast cancer risk and perhaps the effects of preventive interventions among women who have been biopsied for benign breast diseases [16,22].

Our study is limited by the comparatively small sample size, which provides modest power to evaluate associations between TDLU involution and breast cancer subtypes. In addition, because our analyses uses tissues from women with breast cancer, we cannot distinguish whether TDLU morphology influences breast cancer risk or reflects the influence of tumors on the surrounding breast. However, the similarity of the TDLU morphology in samples proximal and distal from the tumor may weaken this argument because other field effects are more pronounced in close proximity to cancer [32]. Another limitation of this case-only study design is that we cannot obtain the relative risks associated with the TDLU involution variables for each tumor subtype. However, acquiring appropriate resources, with the prospective collection of breast tissues prior to cancer diagnosis and sufficient number of cases with CBP tumors developed afterwards, to obtain such risk estimates may be challenging. Finally, we restricted our analysis to younger women to avoid biases related to the ability to observe TDLUs at later ages. Strengths of our study include the population-based design, comprehensive collection of breast cancer risk factors, detailed analysis of marker expression using multiple methods, and availability of cancer and non-cancer tissues collected for research. Our TDLU assessment was subjective; however, our review was masked and reproducible.

## Conclusions

Our findings suggest that the morphology of TDLUs associated with luminal A and CBP cancers varies, which may reflect differences in the pathogenesis of these etiologically distinctive tumors. Confirmation and extension of these findings by performing molecular analyses of TDLUs may provide insights into the pathogenesis of different breast cancer subtypes and/or their secondary effects on peritumoral tissues. Data suggest that CBP cancers may have a high risk of local recurrence [33], which could reflect the presence of residual abnormal epithelium remaining in the breast after initial treatment. Accordingly, future studies to map TDLUs for histopathological and molecular characterization may have value for understanding the pathogenesis of different subtypes of breast cancer and achieving translational goals.

## Abbreviations

95% CI: 95% confidence interval; BMI: body mass index; CBP: core basal phenotype; CK: cytokeratin; DT: distant benign tissue block; EGFR: epidermal growth factor receptor; ER: estrogen receptor; HER2: human epidermal growth factor receptor 2; HRT: hormone replacement therapy; OR: odds ratio; PR: progesterone receptor; PT: peritumoral block; TDLUs: terminal duct lobular units; TMAs: tissue microarrays; TN: triple-negative.

## Competing interests

The authors declare that they have no competing interests.

## Authors' contributions

XRY and MES designed the study. XRY conducted the analysis and drafted the manuscript. JDF contributed to the study design and manuscript preparation. JDF, RTF, JL, BP, LB and MGC contributed to the organization and design of the study. HZ and RMP provided statistical input. SMH contributed to subtyping of breast tumors. MES performed the pathology review and TDLU assessment and contributed to the manuscript preparation. All authors read and approved the final manuscript.

## Supplementary Material

Additional file 1 **Supplementary figures and tables**. Figure S1 and Table S1. Click here for file
