# Supplementary material for: Analysis of terminal duct lobular unit involution in luminal A and basal breast cancers
Source: Breast Cancer Res. 2012 Apr 18;14(2):R64. doi: 10.1186/bcr3170 (PMC3446399; doi:10.1186/bcr3170)
Supplement: Additional file 1 — Supplementary figures and tables. Figure S1 and Table S1. [file bcr3170-S1.DOC]

Supplementary Figure 1. Variation of average number of acini per TDLU and average TDLU diameter by number of TDLUs sampled. Each line represents a breast cancer case with at least 10 TDLUs in either or both non-tumor breast tissue blocks. Data is based on 5000 simulations.


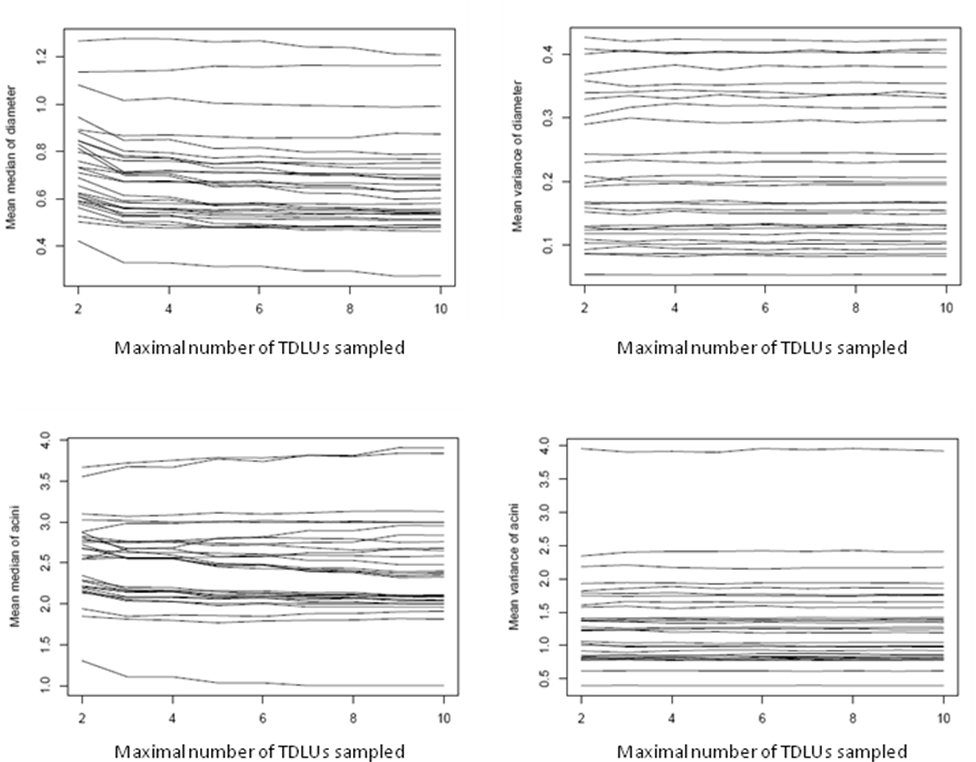


| | Supplementary Table 1. Distribution of breast cancer risk and clinical factors in breast cancer cases (<55 years old) with and without identifiable TDLUs by tumor subtype. | | | | | | | | | | | | | | | | --- | --- | --- | --- | --- | --- | --- | --- | --- | --- | --- | --- | --- | --- | --- | |  |  |  |  |  |  |  |  |  |  |  |  |  |  |  | |  |  | Luminal A | | | | | |  | CBP | | | | | | | Risk and clinical factors |  | Without TDLU | |  | With TDLU | | *P* |  | Without TDLU | |  | With TDLU | | *P* | |  |  | (N= 85) | |  | (N= 232) | |  | (N=19 ) | |  | (N=49 ) | | |  |  | N | % |  | N | % |  | N | % |  | N | % | | Age at diagnosis (year) |  |  |  |  |  |  | 0.72 |  |  |  |  |  |  | 0.14 | | <50 |  | 45 | 52.9 |  | 128 | 55.2 |  |  | 10 | 52.6 |  | 35 | 71.4 |  | | 50-55 |  | 40 | 47.1 |  | 104 | 44.8 |  |  | 9 | 47.4 |  | 14 | 28.6 |  | | Menopausal status |  |  |  |  |  |  | 0.5 |  |  |  |  |  |  | 0.17 | | Pre-menopausal |  | 47 | 55.3 |  | 138 | 59.5 |  |  | 9 | 47.4 |  | 32 | 65.3 |  | | Post-menopausal |  | 38 | 44.7 |  | 94 | 40.5 |  |  | 10 | 52.6 |  | 17 | 34.7 |  | | Education level |  |  |  |  |  |  | 0.72 |  |  |  |  |  |  | 0.31 | | High school or less |  | 55 | 66.3 |  | 148 | 64.1 |  |  | 12 | 63.2 |  | 37 | 75.5 |  | | College degree |  | 28 | 33.7 |  | 83 | 35.9 |  |  | 7 | 36.8 |  | 12 | 24.5 |  | | Age at menarche |  |  |  |  |  |  | 0.98 |  |  |  |  |  |  | 0.58 | | ≤12 |  | 28 | 32.9 |  | 76 | 32.8 |  |  | 5 | 29.4 |  | 21 | 42.9 |  | | 13-14 |  | 42 | 49.4 |  | 117 | 50.4 |  |  | 10 | 58.8 |  | 22 | 44.9 |  | | ≥15 |  | 15 | 17.6 |  | 39 | 16.8 |  |  | 2 | 11.8 |  | 6 | 12.2 |  | | Parity |  |  |  |  |  |  | 0.55 |  |  |  |  |  |  | 0.36 | | No |  | 14 | 16.5 |  | 32 | 13.8 |  |  | 4 | 21.1 |  | 6 | 12.2 |  | | Yes |  | 71 | 83.5 |  | 200 | 86.2 |  |  | 15 | 78.9 |  | 43 | 87.8 |  | | Age at first full-term birth among parous women |  |  |  |  |  |  | 0.38 |  |  |  |  |  |  | 0.27 | | ≤24 |  | 36 | 50.7 |  | 114 | 57.0 |  |  | 9 | 60.0 |  | 25 | 58.1 |  | | 25-30 |  | 28 | 39.4 |  | 61 | 30.5 |  |  | 3 | 20.0 |  | 15 | 34.9 |  | | >30 |  | 7 | 9.9 |  | 25 | 12.5 |  |  | 3 | 20.0 |  | 3 | 7.0 |  | | Breast feeding (month) | |  |  |  |  |  | 0.49 |  |  |  |  |  |  | 0.9 | | Never |  | 23 | 32.4 |  | 57 | 28.5 |  |  | 3 | 20.0 |  | 11 | 25.6 |  | | <12 |  | 40 | 56.3 |  | 109 | 54.5 |  |  | 10 | 66.7 |  | 26 | 60.5 |  | | ≥12 |  | 8 | 11.3 |  | 34 | 17.0 |  |  | 2 | 13.3 |  | 6 | 14.0 |  | | Current BMI |  |  |  |  |  |  | 0.96 |  |  |  |  |  |  | 0.35 | | <25 |  | 37 | 43.5 |  | 102 | 44.0 |  |  | 6 | 31.6 |  | 25 | 51.0 |  | | 25-30 |  | 35 | 41.2 |  | 92 | 39.7 |  |  | 8 | 42.1 |  | 14 | 28.6 |  | | ≥30 |  | 13 | 15.3 |  | 38 | 16.4 |  |  | 5 | 26.3 |  | 10 | 20.4 |  | | Family history of breast cancer in first-degree relatives |  |  |  |  |  |  | 0.57 |  |  |  |  |  |  | 0.44 | | No |  | 79 | 92.9 |  | 210 | 90.9 |  |  | 16 | 84.2 |  | 37 | 75.5 |  | | Yes |  | 6 | 7.1 |  | 21 | 9.1 |  |  | 3 | 15.8 |  | 12 | 24.5 |  | | Smoking |  |  |  |  |  |  | 0.66 |  |  |  |  |  |  | 0.15 | | Never |  | 28 | 32.9 |  | 64 | 27.7 |  |  | 8 | 42.1 |  | 16 | 32.7 |  | | Past |  | 18 | 21.2 |  | 51 | 22.1 |  |  | 1 | 5.3 |  | 13 | 26.5 |  | | Current |  | 39 | 45.9 |  | 116 | 50.2 |  |  | 10 | 52.6 |  | 20 | 40.8 |  | | Alcohol drinking |  |  |  |  |  |  | 0.06 |  |  |  |  |  |  | 0.09 | | Never |  | 56 | 65.9 |  | 122 | 52.8 |  |  | 11 | 57.9 |  | 29 | 59.2 |  | | Past |  | 8 | 9.4 |  | 19 | 8.2 |  |  | 5 | 26.3 |  | 4 | 8.2 |  | | Current |  | 21 | 24.7 |  | 90 | 39.0 |  |  | 3 | 15.8 |  | 16 | 32.7 |  | | Tumor size |  |  |  |  |  |  | 0.08 |  |  |  |  |  |  | 0.26 | | ≤ 2 cm |  | 59 | 69.4 |  | 135 | 58.4 |  |  | 9 | 47.4 |  | 16 | 32.7 |  | | > 2 cm |  | 26 | 30.6 |  | 96 | 41.6 |  |  | 10 | 52.6 |  | 33 | 67.3 |  | | Axillary node metastases |  |  |  |  |  |  | 0.12 |  |  |  |  |  |  | 0.46 | | Negative |  | 56 | 65.9 |  | 128 | 56.1 |  |  | 10 | 52.6 |  | 30 | 62.5 |  | | Positive |  | 29 | 34.1 |  | 100 | 43.9 |  |  | 9 | 47.4 |  | 18 | 37.5 |  | | Histology |  |  |  |  |  |  | 0.8 |  |  |  |  |  |  | 0.26 | | Ductal |  | 55 | 64.7 |  | 136 | 58.6 |  |  | 15 | 78.9 |  | 42 | 85.7 |  | | Lobular |  | 14 | 16.5 |  | 44 | 19.0 |  |  | 1 | 5.3 |  | 0 | 0.0 |  | | Mixed |  | 9 | 10.6 |  | 31 | 13.4 |  |  | 1 | 5.3 |  | 5 | 10.2 |  | | Other |  | 7 | 8.2 |  | 21 | 9.1 |  |  | 2 | 10.5 |  | 2 | 4.1 |  | | Tumor grade |  |  |  |  |  |  | 0.9 |  |  |  |  |  |  | 0.27 | | Well differentiated |  | 23 | 27.1 |  | 69 | 29.7 |  |  | 0 | 0.0 |  | 0 | 0.0 |  | | Moderately differentiated |  | 52 | 61.2 |  | 137 | 59.1 |  |  | 3 | 15.8 |  | 14 | 28.6 |  | | Poorly differentiated |  | 10 | 11.8 |  | 26 | 11.2 |  |  | 16 | 84.2 |  | 35 | 71.4 |  | |  |  |  |  |  |  |  |  |  |  |  |  |  |  |  | | P values were obtained by comparing cases with and without TDLUs using likelihood ratio test for differences in frequencies. | | | | | | | | | | | | | | | |
| --- | --- | --- | --- | --- | --- | --- | --- | --- | --- | --- | --- | --- | --- | --- | --- | --- | --- | --- | --- | --- | --- | --- | --- | --- | --- | --- | --- | --- | --- | --- | --- | --- | --- | --- | --- | --- | --- | --- | --- | --- | --- | --- | --- | --- | --- | --- | --- | --- | --- | --- | --- | --- | --- | --- | --- | --- | --- | --- | --- | --- | --- | --- | --- | --- | --- | --- | --- | --- | --- | --- | --- | --- | --- | --- | --- | --- | --- | --- | --- | --- | --- | --- | --- | --- | --- | --- | --- | --- | --- | --- | --- | --- | --- | --- | --- | --- | --- | --- | --- | --- | --- | --- | --- | --- | --- | --- | --- | --- | --- | --- | --- | --- | --- | --- | --- | --- | --- | --- | --- | --- | --- | --- | --- | --- | --- | --- | --- | --- | --- | --- | --- | --- | --- | --- | --- | --- | --- | --- | --- | --- | --- | --- | --- | --- | --- | --- | --- | --- | --- | --- | --- | --- | --- | --- | --- | --- | --- | --- | --- | --- | --- | --- | --- | --- | --- | --- | --- | --- | --- | --- | --- | --- | --- | --- | --- | --- | --- | --- | --- | --- | --- | --- | --- | --- | --- | --- | --- | --- | --- | --- | --- | --- | --- | --- | --- | --- | --- | --- | --- | --- | --- | --- | --- | --- | --- | --- | --- | --- | --- | --- | --- | --- | --- | --- | --- | --- | --- | --- | --- | --- | --- | --- | --- | --- | --- | --- | --- | --- | --- | --- | --- | --- | --- | --- | --- | --- | --- | --- | --- | --- | --- | --- | --- | --- | --- | --- | --- | --- | --- | --- | --- | --- | --- | --- | --- | --- | --- | --- | --- | --- | --- | --- | --- | --- | --- | --- | --- | --- | --- | --- | --- | --- | --- | --- | --- | --- | --- | --- | --- | --- | --- | --- | --- | --- | --- | --- | --- | --- | --- | --- | --- | --- | --- | --- | --- | --- | --- | --- | --- | --- | --- | --- | --- | --- | --- | --- | --- | --- | --- | --- | --- | --- | --- | --- | --- | --- | --- | --- | --- | --- | --- | --- | --- | --- | --- | --- | --- | --- | --- | --- | --- | --- | --- | --- | --- | --- | --- | --- | --- | --- | --- | --- | --- | --- | --- | --- | --- | --- | --- | --- | --- | --- | --- | --- | --- | --- | --- | --- | --- | --- | --- | --- | --- | --- | --- | --- | --- | --- | --- | --- | --- | --- | --- | --- | --- | --- | --- | --- | --- | --- | --- | --- | --- | --- | --- | --- | --- | --- | --- | --- | --- | --- | --- | --- | --- | --- | --- | --- | --- | --- | --- | --- | --- | --- | --- | --- | --- | --- | --- | --- | --- | --- | --- | --- | --- | --- | --- | --- | --- | --- | --- | --- | --- | --- | --- | --- | --- | --- | --- | --- | --- | --- | --- | --- | --- | --- | --- | --- | --- | --- | --- | --- | --- | --- | --- | --- | --- | --- | --- | --- | --- | --- | --- | --- | --- | --- | --- | --- | --- | --- | --- | --- | --- | --- | --- | --- | --- | --- | --- | --- | --- | --- | --- | --- | --- | --- | --- | --- | --- | --- | --- | --- | --- | --- | --- | --- | --- | --- | --- | --- | --- | --- | --- | --- | --- | --- | --- | --- | --- | --- | --- | --- | --- | --- | --- | --- | --- | --- | --- | --- | --- | --- | --- | --- | --- | --- | --- | --- | --- | --- | --- | --- | --- | --- | --- | --- | --- | --- | --- | --- | --- | --- | --- | --- | --- | --- | --- | --- | --- | --- | --- | --- | --- | --- | --- | --- | --- | --- | --- | --- | --- | --- | --- | --- | --- | --- | --- | --- | --- | --- | --- | --- | --- | --- | --- | --- | --- | --- | --- | --- | --- | --- | --- | --- | --- | --- | --- | --- | --- | --- | --- | --- | --- | --- | --- | --- | --- | --- | --- | --- | --- | --- | --- | --- | --- | --- | --- | --- | --- | --- | --- | --- | --- | --- | --- | --- | --- | --- | --- | --- | --- | --- | --- | --- | --- | --- | --- | --- | --- | --- | --- | --- | --- | --- | --- | --- | --- | --- | --- | --- | --- | --- | --- | --- | --- | --- | --- | --- | --- | --- | --- | --- | --- | --- | --- | --- | --- | --- | --- | --- | --- | --- | --- | --- | --- | --- | --- | --- | --- | --- | --- | --- | --- | --- | --- | --- | --- | --- | --- | --- | --- | --- | --- | --- | --- | --- | --- | --- | --- | --- | --- | --- | --- | --- | --- | --- | --- | --- | --- | --- | --- | --- | --- | --- | --- | --- | --- | --- | --- | --- | --- | --- | --- | --- | --- | --- | --- | --- | --- | --- | --- | --- | --- | --- | --- | --- | --- | --- | --- | --- | --- | --- | --- | --- | --- | --- | --- | --- | --- | --- | --- | --- | --- | --- | --- | --- | --- | --- | --- | --- | --- | --- | --- | --- | --- | --- | --- | --- | --- | --- | --- | --- | --- | --- | --- | --- | --- | --- | --- | --- | --- | --- | --- | --- | --- | --- | --- | --- | --- | --- | --- | --- | --- | --- | --- | --- | --- | --- | --- | --- | --- | --- | --- | --- | --- | --- | --- | --- | --- | --- | --- | --- | --- | --- | --- | --- | --- | --- | --- | --- | --- | --- | --- | --- | --- | --- | --- | --- | --- | --- | --- | --- | --- | --- | --- | --- | --- | --- | --- | --- | --- | --- | --- | --- | --- | --- | --- | --- | --- | --- | --- | --- | --- | --- | --- | --- | --- | --- | --- | --- | --- | --- | --- | --- | --- | --- | --- | --- | --- | --- | --- | --- | --- | --- | --- | --- | --- | --- | --- | --- | --- | --- | --- | --- | --- | --- | --- | --- | --- | --- | --- | --- | --- | --- | --- | --- | --- | --- | --- | --- | --- | --- | --- | --- | --- | --- | --- | --- | --- | --- | --- | --- | --- | --- | --- | --- | --- | --- | --- | --- | --- | --- | --- | --- | --- | --- | --- | --- | --- | --- | --- | --- | --- | --- | --- | --- | --- | --- | --- | --- | --- | --- | --- | --- | --- | --- |
